# Long-Term Survival in a Dog After Adrenalectomy and Splenectomy for Two Different Malignancies with Portal Vein Involvement

**DOI:** 10.3390/ani15213159

**Published:** 2025-10-30

**Authors:** Seung-Hyun Kim, Jun-Gyu Park, Jang-Han Yoon, Yeong-Bin Baek, Sang-Ik Park

**Affiliations:** 1 KSH Surgical Animal Medical Center, Gwangju 61617, Republic of Korea; trazet08@gmail.com (S.-H.K.); tomcoma1@gmail.com (J.-H.Y.); 2 Department of Veterinary Zoonotic Diseases, College of Veterinary Medicine, Chonnam National University, Gwangju 61186, Republic of Korea; kingsalt@jnu.ac.kr; 3 Department of Veterinary Pathology, College of Veterinary Medicine, Chonnam National University, Gwangju 61186, Republic of Korea; 4 Department of Veterinary Pathology, College of Veterinary Medicine and BK21 FOUR Program, Chonnam National University, Gwangju 61186, Republic of Korea

**Keywords:** primary tumors, multimodal surgery, pheochromocytoma, B-cell lymphoma, portal vein involvement

## Abstract

**Simple Summary:**

Cancer is one of the most serious health problems in dogs, and occasionally, two different types of cancer can develop at the same time. This report describes a dog diagnosed with two separate tumors—one in the adrenal gland and another in the spleen. The adrenal tumor was closely attached to a major blood vessel (the portal vein), a condition that has not been previously reported in veterinary medicine. Despite the technical difficulty, the veterinary team successfully removed the adrenal gland, the spleen, and nearby lymph nodes while preserving the vessel. The dog recovered uneventfully and remained healthy for three years after surgery without additional treatment. This case illustrates that careful surgical planning and meticulous tumor removal can lead to long-term remission even in complex oncologic situations.

**Abstract:**

Concurrent occurrence of two independent primary malignancies in a single dog is rare and presents diagnostic and surgical challenges. A 9-year-old neutered male Cocker Spaniel was diagnosed with adrenal pheochromocytoma and splenic diffuse large B-cell lymphoma. Abdominal imaging revealed two distinct masses. Surgical management included adrenalectomy, splenectomy, mesenteric lymphadenectomy, and excision of a small mass adherent to the portal vein adventitia. Histopathology confirmed two separate malignancies, with chromogranin A positivity supporting pheochromocytoma and CD20 positivity confirming B-cell lymphoma. No additional metastatic lesions were identified, and the portal vein-associated mass was considered an isolated lesion closely adherent to the vessel wall, with its exact pathogenesis remaining uncertain. To the authors’ knowledge, this represents the first veterinary report describing adrenal pheochromocytoma with portal vein involvement successfully managed by surgical removal. The patient recovered well and remained disease-free for three years without adjuvant therapy. This case emphasizes that, even in technically demanding situations, meticulous surgical planning and comprehensive oncologic assessment can achieve durable remission and inform future approaches to complex veterinary cancers.

## 1. Introduction

Veterinary oncologists are increasingly encountering patients that present with two synchronous, independent primary malignancies—distinct neoplasms arising in separate organs, each confirmed histopathologically and not attributable to inter-lesional metastatic spread [1,2]. Although such presentations are well documented in human oncology, reports in small animals remain relatively uncommon, and most veterinary publications to date consist of isolated case descriptions of coexisting primary tumors of different histogenesis [3,4]. Documenting such rare cases provides practical insights into diagnostic differentiation, treatment planning, and postoperative expectations

Among small-animal neoplasms, primary adrenal tumors are relatively rare, representing only 0.17–0.76% of all canine tumors [5]. The most common tumor types are adrenocortical adenoma, adrenocortical carcinoma, and pheochromocytoma [6]. Clinical presentations span incidental imaging findings to hormonally active disease with hemodynamic instability, particularly in pheochromocytoma. When surgery is clinically indicated, adrenalectomy is a standard procedure that nonetheless poses nontrivial technical and physiologic challenges due to the intimate relationship with major vessels and the risk of catecholamine surges [7,8]. In appropriately selected cases, surgical removal remains the intervention with curative intent, and more advanced vascular techniques have broadened eligibility in patients with caudal vena cava (CVC) involvement, including cavotomy or venectomy in specialized settings [8,9].

By contrast, involvement of adrenal tumors with the portal vein (PV) is exceptionally rare, and to our knowledge, no prior veterinary reports have documented such cases. This rarity underscores the clinical significance of the present case. In human hepatobiliary oncology, portal vein involvement has been described in association with hepatocellular carcinoma, where aggressive surgical approaches, including venotomy or partial venectomy, are sometimes employed to improve outcomes despite considerable technical challenges and perioperative risk [10].

Splenic masses are common in older dogs, but primary splenic lymphoma is rare, accounting for only 0.02–0.1% of canine tumors [11]. Histologic subtypes of splenic lymphoma follow the World Health Organization (WHO) classification, including diffuse large B-cell lymphoma (DLBCL), marginal zone lymphoma (MZL), mantle cell lymphoma (MCL), follicular lymphoma, peripheral T-cell lymphoma, and NK-cell lymphoma [12]. Among these, DLBCL is most common and typically more aggressive, whereas MZL follows a more indolent course.

Splenectomy remains the primary surgical intervention, especially in emergent situations involving rupture or hemoabdomen, and serves both therapeutic and diagnostic purposes [13]. While splenectomy alone confers limited survival benefit, integration with systemic chemotherapy (CHOP-based protocols) significantly prolongs survival, with median durations exceeding one year in responsive cases [14]. In human lymphoma, splenectomy has historically been reserved for diagnostic or palliative indications, but recent data suggest that patients undergoing splenectomy in addition to systemic therapy may achieve improved remission and survival compared with systemic therapy alone [15].

Beyond organ resection, lymphadenectomy has emerged as an important adjunct to comprehensive surgical management. In canine oncology, removal of metastatic regional lymph nodes has been associated with improved progression-free and overall survival, particularly in mast cell tumors, where dogs undergoing lymphadenectomy showed significantly longer disease control intervals [16]. Similarly, in human gastrointestinal oncology, systematic mesenteric lymphadenectomy—as in complete mesocolic excision (CME) or D3 dissection—has demonstrated reductions in locoregional recurrence and improvements in long-term survival [17,18]. By analogy, mesenteric lymphadenectomy in canine splenic lymphoma may help reduce relapse and extend disease-free intervals, particularly when integrated into multimodal surgical strategies.

The present report describes a dog with two independent primary malignancies—an adrenal pheochromocytoma associated with a small lesion adherent to the portal vein adventitia and a splenic B-cell lymphoma. The patient underwent adrenalectomy, excision of the portal vein-adherent mass, splenectomy, and mesenteric lymphadenectomy. The long-term, recurrence-free outcome demonstrates that although these procedures are technically demanding in small animal surgery, their prognostic impact can be substantial when successful. This report aims to present the diagnostic findings, surgical management, and clinical outcome of this rare tumor combination, emphasizing the anatomical and technical considerations involved.

## 2. Case Description

A 9-year-old neutered male Cocker Spaniel was presented with a 7-day history of severe anorexia, lethargy, and multiple episodes of vomiting and diarrhea. At the first physical examination, the patient weighed 9.2 kg and was classified as ASA physical status III. The body condition score (BCS) was approximately 6 out of 9, indicating a slightly overweight condition. Cardiac auscultation revealed a mild systolic murmur (grade 1/6), with no other remarkable physical abnormalities observed. However, the blood pressure at presentation was markedly elevated, with a systolic arterial pressure (SAP) of approximately 175 mmHg and a mean arterial pressure (MAP) of 120 mmHg. The patient was hospitalized immediately, and a complete diagnostic work-up was initiated, including complete blood count (CBC), serum biochemistry, electrolyte analysis, thoracic radiography, and abdominal ultrasonography.

**Figure 1:**
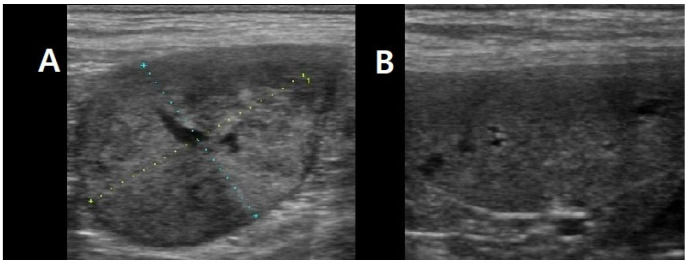
Ultrasonographic images of abdominal tumors. (**A**) An unidentified left retroperitoneal mass (approximately 3 × 2 cm in diameter). (**B**) The spleen.

Laboratory analyses revealed a marked increase in canine C-reactive protein (cCRP, 95.4 mg/L; reference range: <20 mg/L) and canine pancreatic lipase (cPL, 975 μg/L; reference range: <200 μg/L). The complete blood count (CBC) demonstrated moderate leukocytosis (23.5 × 10^9^/L; reference range: 6.0–17.0 × 10^9^/L) characterized by neutrophilia (14.8 × 10^9^/L; reference range: 3.0–11.5 × 10^9^/L). All other hematologic and biochemical parameters were within normal limits. Thoracic radiography and abdominal ultrasonography identified an uncharacterized left retroperitoneal mass measuring approximately 3 × 2 cm in diameter, in addition to a splenic mass (Figure 1).

**Figure 2:**
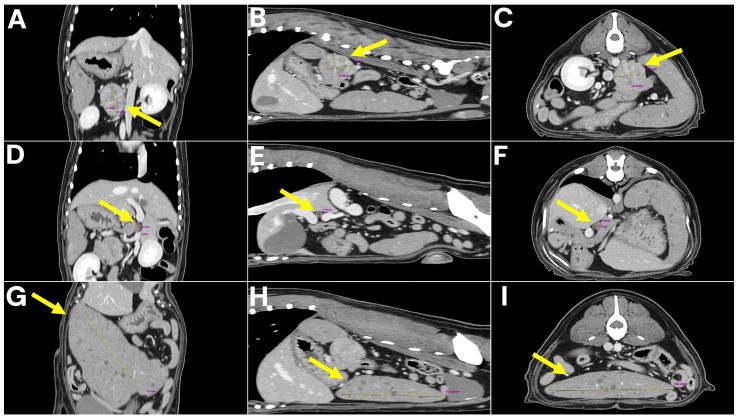
CT images of abdominal tumors. (**A**–**C**) Left adrenal gland mass (4 × 3 × 3 cm) shown in coronal, sagittal, and axial views, with arrows indicating the mass. (**D**–**F**) Lesion adherent to the wall of the hepatic portal vein (7 mm) visualized in coronal, sagittal, and axial views, with arrows marking the site of vascular attachment. (**G**–**I**) Splenic nodular masses demonstrated in coronal, sagittal, and axial views, with arrows identifying the nodular masses.

To further characterize the lesions and assess for metastatic disease, computed tomography (CT) was performed. No pulmonary metastasis was detected; however, multiple intra-abdominal masses with suspected metastatic lesions were observed. The retroperitoneal mass was delineated as a 4 × 3 × 3 cm lesion arising from the left adrenal gland, consistent with a tentative diagnosis of adrenal tumor. The spleen was enlarged and contained multiple nodular masses, while the mesenteric lymph nodes were markedly enlarged. In addition, a 7 mm lesion adherent to the wall of the hepatic portal vein (extraluminal mass) was detected and initially interpreted as a possible metastatic lesion (Figure 2).

Also, prior to surgery, a urine cortisol-to-creatinine ratio (UCCR) test was performed to assess adrenal function. The UCCR was 19, which was within the normal range (reference value: ≤34) and did not suggest hypercortisolism. However, urinary catecholamine metabolite analysis revealed elevated concentrations of metanephrine (15.32 nmol/L; reference range: 0.3–4.1 nmol/L) and normetanephrine (42.12 nmol/L; reference range: 0.8–5.1 nmol/L), suggesting that a pheochromocytoma, an adrenal medulla tumor, was more likely than an adrenocortical tumor. Given these findings, definitive surgical resection was determined to be the most appropriate therapeutic option, and an exploratory laparotomy was undertaken. Preoperative evaluation further identified systemic hypertension, with an initial systolic arterial pressure (SAP) of approximately 180 mmHg and a mean arterial pressure (MAP) of 130 mmHg. To achieve hemodynamic stabilization prior to surgery, a continuous intravenous nitroglycerin infusion (0.3 µg/kg/min) was administered, resulting in a reduction in arterial pressures to a stable SAP of 125 mmHg and MAP of 80 mmHg at the time of surgical induction. The patient was maintained on intravenous Hartmann’s solution throughout the perioperative period.

A 24-gauge intravenous catheter was placed in the right cephalic vein, and pre-anesthetic medication included remifentanil (1 µg/kg, IV). After 2 min of preoxygenation, anesthesia was induced with alfaxalone (2 mg/kg to effect, IV). Endotracheal intubation was achieved using a 4 mm internal diameter endotracheal tube, with the cuff inflated to prevent air leaks at 20 cm H_2_O. The patient was maintained on a rebreathing system with oxygen at 1 L/min and isoflurane (1%), in combination with a remifentanil constant rate infusion (0.2 µg/kg/min). Pressure-regulated volume control ventilation (PRVC) was employed, with settings adjusted to achieve a tidal volume of 10 mL/kg predicted body weight and partial pressure of end-tidal CO_2_ maintained between 35 and 45 mmHg.

**Figure 3:**
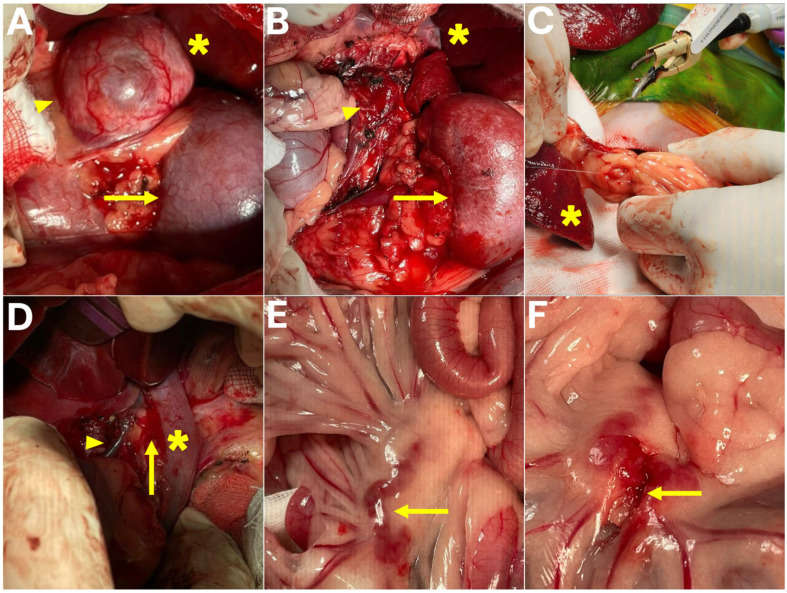
Intraoperative images of surgical procedures. (**A**) Left adrenal mass prior to adrenalectomy (asterisk, liver; arrowhead, adrenal tumor; arrow, left kidney). (**B**) Surgical field following adrenalectomy (asterisk, liver; arrowhead, adrenalectomy site; arrow, left kidney). (**C**) Spleen prior to splenectomy (asterisk, spleen under removal). (**D**) PV-associated mass excision performed as a wall-associated tumor excision, preserving the vascular lumen (asterisk, portal vein; arrowhead, common bile duct; arrow, portal vein adventitial tumor excision site). (**E**) Mesenteric lymph node prior to lymphadenectomy (arrow, mesenteric lymph node). (**F**) Surgical field following lymphadenectomy (arrow, lymphadenectomy site).

The patient was positioned in dorsal recumbency and prepared for aseptic surgery. A ventral midline laparotomy was performed to provide access to the abdominal cavity. Upon exploration, the left adrenal gland was identified cranial to the left kidney, with no evidence of invasion into the caudal vena cava (Figure 3). Careful dissection was carried out to isolate the adrenal gland from surrounding tissues. The abdominal phrenic vein, adrenal artery, and adrenal vein were meticulously identified, ligated, and transected using a combination of Thunderbeat, a fine bipolar tip, and conventional suture ligation. Special attention was directed toward preserving the adjacent renal vessels and avoiding hemorrhage, and following complete vascular isolation, the left adrenal gland and associated mass were excised en bloc.

An excision of a mass adherent to the portal vein adventitia was performed; however, unlike conventional intraluminal thrombectomy or venectomy, this procedure was classified as a portal vein wall-associated tumor excision, preserving the integrity of the vascular lumen and avoiding venotomy or vascular reconstruction. The lesion was confirmed intraoperatively to be adherent to the external wall of the extrahepatic portal vein rather than occupying the lumen. Dissection was performed with extreme caution using Thunderbeat, a fine bipolar tip, and sterile cotton swabs to achieve both blunt and sharp separation of the tumor tissue from the vessel wall. Partial removal of the adventitial layer was necessary; however, there was no evidence of intraluminal invasion, and vascular reconstruction was not required. To prevent catastrophic hemorrhage, dissection was carried out in a stepwise fashion with continuous inspection of the portal vein wall.

In addition, the splenic mass was excised. A splenectomy was performed using Thunderbeat in combination with suture ligation for vascular control, with each hilar vessel secured sequentially to prevent bleeding and to avoid injury to the pancreatic tail. The spleen was removed in its entirety without complications. Furthermore, an enlarged mesenteric lymph node was identified and carefully dissected and excised, with fine bipolar coagulation and atraumatic handling employed to prevent hemorrhage or bowel injury while maintaining mesenteric blood supply.

The surgical field was thoroughly inspected, and hemostasis was confirmed. The abdominal cavity was irrigated with warm sterile saline, and no drains were placed. Routine three-layer abdominal closure was performed.

The patient remained hospitalized in the intensive care unit (ICU) for seven days following surgery. Postoperative management included intravenous antibiotics, opioid analgesia, an antithrombotic agent, a gastrointestinal protectant, an antiemetic, and fluid therapy. Specifically, ampicillin/sulbactam (20 mg/kg IV, TID), metronidazole (10 mg/kg IV, BID), and marbofloxacin (2 mg/kg IV, SID) were administered as antimicrobial therapy. Analgesia was provided with hydromorphone (0.02 mg/kg/hr IV CRI) for approximately two days postoperatively. Clopidogrel (1 mg/kg PO, SID) was given as an antithrombotic agent, while esomeprazole (1 mg/kg IV, SID) was administered for gastrointestinal protection. To prevent postoperative nausea and vomiting, maropitant (1 mg/kg IV, SID) was used. A balanced crystalloid solution was continuously infused to maintain hydration and electrolyte balance throughout the postoperative period. Vital parameters, including blood pressure, heart rate, respiratory rate, and body temperature, were monitored more than six times daily. In addition, routine blood tests—including complete blood count (CBC), serum biochemistry (liver, kidney, gallbladder, and pancreas parameters), electrolytes, C-reactive protein (CRP), and cortisol levels—were performed every other day. Cortisol concentrations remained within the normal range throughout the postoperative period, and therefore glucocorticoid supplementation was not indicated. From the second postoperative day, the patient demonstrated recovery of vitality, including the return of spontaneous appetite. Fortunately, no major complications such as Addisonian crisis or thromboembolic events occurred during the postoperative period, and the patient was discharged in good health.

**Figure 4:**
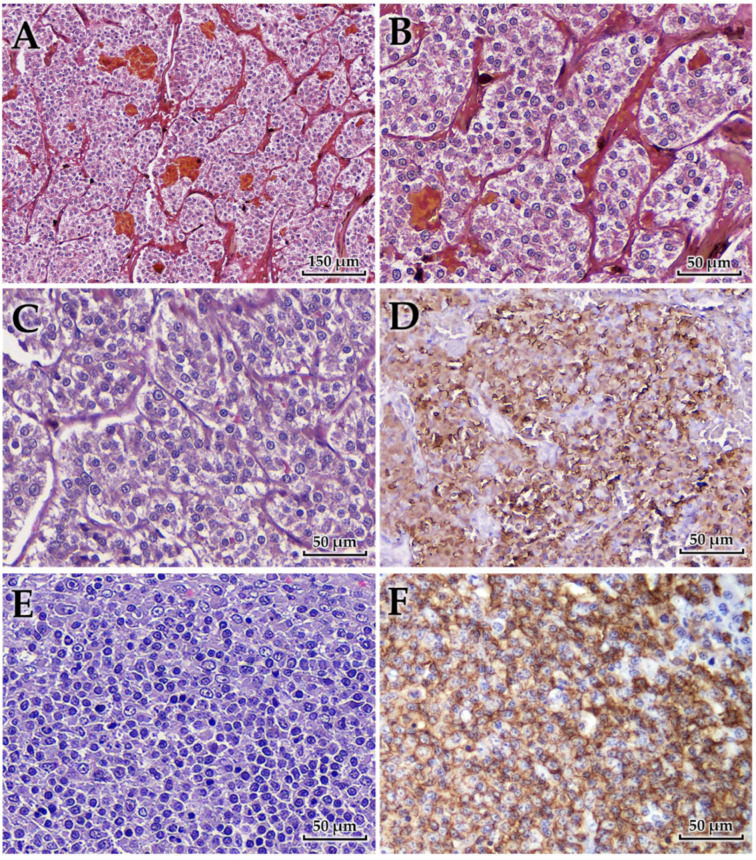
Microscopic features of the adrenal tumor, PV wall involvement, and splenic lymphoma. (**A**) Primary adrenal pheochromocytoma showing tumor nests separated by thin fibrovascular septa, with areas of congestion and multifocal hemorrhage. (**B**) Higher magnification of adrenal tumor cells exhibiting lightly eosinophilic, finely granular cytoplasm; neoplastic cells range from polyhedral to pleomorphic forms. (**C**) Tumor tissue adherent to the wall of the portal vein, morphologically consistent with the adrenal pheochromocytoma, indicating secondary vascular wall involvement. (**D**) Immunohistochemical staining for chromogranin A demonstrating strong labeling of chromaffin granules within adrenal tumor cells. (**E**) Splenic diffuse large B-cell lymphoma composed of large sheets of centroblasts and immunoblasts with euchromatic nuclei, vesicular or coarsely granular chromatin, and prominent amphophilic nucleoli. (**F**) Immunohistochemistry of splenic lymphoma showing positive labeling for CD20 in neoplastic B cells.

Histopathological examination revealed distinct lesions in the adrenal gland, portal vein, and spleen (Figure 4). The adrenal tumor was composed of polyhedral to pleomorphic neoplastic cells with lightly eosinophilic, finely granular, and often indistinct cytoplasm. The cells were arranged in small lobules or nests along sinusoidal vessels, subdivided by fine fibrovascular septa and capillaries. Areas of coagulative necrosis and hemorrhage were observed throughout the lesions. Immunohistochemically, the neoplastic cells were strongly positive for chromogranin A but negative for cytokeratin and vimentin, consistent with a diagnosis of pheochromocytoma of medullary origin.

Tumor tissue was found adherent to the wall of the PV, morphologically indistinguishable from the adrenal lesion. This finding was interpreted as secondary involvement of the portal vein wall through metastatic spread, rather than direct extension from the adrenal gland. Histologically, the lesion showed pheochromocytoma cells. The tumor cells exhibited the same pleomorphic, polyhedral morphology with finely granular eosinophilic cytoplasm as observed in the adrenal mass.

The spleen showed diffuse replacement of the architecture by large lymphoid cells expanding within the white pulp and compressing the red pulp. Sheets of neoplastic cells formed a characteristic “starry-sky” pattern due to interspersed tingible-body macrophages. The cells consisted of centroblasts with vesicular to coarsely granular chromatin and multiple amphophilic nucleoli, and immunoblasts with a single, centrally located nucleolus. Mitotic figures were often observed. Immunohistochemically, the neoplastic cells were positive for CD20 and negative for CD3, confirming B-cell lineage and supporting the diagnosis of high-grade diffuse large B-cell lymphoma (DLBCL) [14,19]. Examination of mesenteric lymph nodes revealed reactive follicular hyperplasia without evidence of neoplastic infiltration, indicating the absence of metastasis.

## 3. Discussion

Histopathological examination in the present case confirmed two malignant tumors—an adrenal pheochromocytoma associated with a small lesion adherent to the PV adventitia and splenic DLBCL—along with reactive, non-neoplastic changes in mesenteric lymph nodes. As these tumors were topographically separate and displayed distinct histogenesis, they were interpreted as two independent primary malignancies rather than metastatic lesions. Although the precise tumorigenesis could not be defined due to limited patient history, this case adds to the sparse veterinary literature on concurrent primary tumors in dogs.

A central clinical challenge in such presentations lies in distinguishing independent primary tumors from metastatic spread, as this distinction directly affects staging, treatment planning, and prognosis [1,2]. In human oncology, multiple synchronous malignancies are increasingly recognized, whereas reports in veterinary medicine remain sparse. Documenting such cases is therefore valuable, as it contributes to refining diagnostic and therapeutic approaches in veterinary oncology.

Adrenalectomy remains one of the most technically demanding abdominal surgeries in dogs, complicated by risks such as hemorrhage, hemodynamic instability, and thromboembolism [20,21]. Nevertheless, when performed successfully, it offers the only curative option and significantly improves survival, even in cases with vascular invasion [9,22].

The choice between laparoscopic adrenalectomy (LA) and open adrenalectomy (OA) has been increasingly discussed. While LA offers smaller incisions and faster recovery, its limitations in visualization and instrument maneuverability increase the risk of capsular rupture, tumor spillage, and incomplete excision. Reported conversion rates to open surgery and capsular penetration underscore these risks [23,24]. In contrast, OA provides superior exposure and vascular control, which is particularly critical for large or vascularly associated adrenal tumors [25,26]. In this case, OA was selected to ensure safe and controlled removal of the adrenal tumor and its associated adventitial lesion.

Vascular involvement in canine adrenal tumors most often affects the CVC, with reported frequencies up to 50% in pheochromocytomas [27]. By contrast, association with the portal vein is extremely rare. To our knowledge, no prior veterinary reports have described a pheochromocytoma accompanied by a lesion adherent to the PV adventitia. The differential interpretation of this finding is complex. Although direct extension from the adrenal gland is theoretically possible, it would typically be accompanied by local tissue invasion, perivascular fibrosis, or necrosis [28,29]—findings not evident in this case. Conversely, the pattern observed here, in which a small nodular lesion was confined to the PV adventitia without transmural invasion, may suggest secondary implantation via microscopic lymphatic or vascular spread. Both interpretations are plausible, and in the revised manuscript, we have discussed these alternative possibilities to reflect a balanced pathological assessment [30]. Future accumulation of similar cases, ideally including en bloc resection specimens and serial histologic sectioning of perivascular tissues, will be necessary to clarify the exact mechanism of PV involvement.

In human hepatobiliary oncology, resections addressing PV wall invasion, such as partial venectomy or local tumor excision, have been described in hepatocellular carcinoma and are associated with improved outcomes despite high technical demand and perioperative risk [10]. In veterinary cases, experience remains limited, but available evidence suggests that careful excision of a mass adherent to the PV adventitia—without venotomy or venectomy—can be safely achieved and may yield favorable outcomes [31]. In our case, the procedure preserved the vascular lumen, but histologic margin assessment was inherently limited due to the need to maintain vessel integrity, which represents a diagnostic and surgical constraint that should be acknowledged in future cases.

Lymphadenectomy in oncology serves both diagnostic and therapeutic roles. In dogs, removal of metastatic lymph nodes in mast cell tumors has been shown to prolong disease-free and overall survival [16]. In human gastrointestinal oncology, systematic mesenteric lymphadenectomy, such as complete mesocolic excision or D3 dissection, improves locoregional control and long-term outcomes [17,18]. In this case, mesenteric lymphadenectomy confirmed the absence of metastasis and likely reduced the risk of microscopic residual disease, contributing to the favorable long-term outcome.

Overall, this case underscores three main points. First, although rare, concurrent independent malignancies can occur in dogs and should be carefully distinguished from metastatic disease. Second, standard adrenalectomy combined with excision of PV-adherent lesions, when performed with adequate hemodynamic preparation and intraoperative control, can be safely achieved and may yield durable oncologic outcomes despite technical challenges. Third, regional lymphadenectomy remains an important adjunct for both staging and disease control, even when metastasis is not grossly evident.

The patient’s long-term survival—three years without recurrence or adjuvant therapy—suggests that comprehensive surgical management, when meticulously planned and executed, can shift the prognosis of complex malignancies from palliative to potentially curative. Nevertheless, the inability to fully evaluate surgical margins, particularly in the PV-associated lesion, represents a limitation of this case and should be interpreted with caution when considering long-term oncologic outcomes. Future reports combining detailed histopathological mapping and standardized terminology will be critical to improve understanding and treatment of such rare but clinically significant presentations.

## 4. Conclusions

This case report describes the successful surgical management of two independent primary malignancies—an adrenal pheochromocytoma associated with a small lesion adherent to the portal vein adventitia and a splenic diffuse large B-cell lymphoma—in a dog. Adrenalectomy, excision of the portal vein-associated lesion, splenectomy, and mesenteric lymphadenectomy were performed safely, resulting in long-term, recurrence-free survival without adjuvant therapy.

Although surgery involving vascular structures remains technically challenging, this case demonstrates that careful surgical planning and preservation of vascular integrity can yield favorable outcomes. The rarity of portal vein-associated lesions in adrenal tumors highlights the clinical relevance of detailed anatomical assessment and tailored surgical management. Overall, this case contributes valuable practical insight into the operative and pathological considerations for complex, multi-organ neoplasms in small animal oncology.

## Data Availability

All data are contained within the article.

## References

[1] Zhai C.Y., Cai Y.L., Lou F., Liu Z., Xie J.S., Zhou X.Y., Wang Z.G., Fang Y., Pan H.M., Han W.D. (2018). Multiple Primary Malignant Tumors—A Clinical Analysis of 15,321 Patients with Malignancies at a Single Center in China. J. Cancer.

[2] Lv M., Zhang X., Shen Y., Wang F., Yang J., Wang B., Chen Z., Li P., Zhang X., Li S. (2017). Clinical analysis and prognosis of synchronous and metachronous multiple primary malignant tumors. Medicine.

[3] Kim S.H., Baek Y.B., Park S.I. (2024). Canine multiple primary tumours: Mammary tubular carcinoma, uterine leiomyosarcoma, and facial sebaceous epithelioma. Vet. Med..

[4] Kim S.H., Baek Y.B., Park S.I. (2024). Concurrent primary splenic lymphoma and mammary gland tumour with polycystic ovaries in a dog. Vet. Med..

[5] Carafi O.A., Imlau M., Dalla Serra G., Puggioni A., Shorten E., Cloack B., Hoey S. (2024). Ex vivo MRI and histological comparison of the canine adrenal glands. Vet. Radiol. Ultrasound.

[6] Barrera J.S., Bernard F., Ehrhart E.J., Withrow S.J., Monnet E. (2013). Evaluation of risk factors for outcome associated with adrenal gland tumors with or without invasion of the caudal vena cava and treated via adrenalectomy in dogs: 86 cases (1993–2009). Javma-J. Am. Vet. Med. A.

[7] Lipscomb V.J. (2019). Surgical management of an adrenal gland tumor that had extended into the thoracic portion of the caudal vena cava in a dog. Javma-J. Am. Vet. Med. A.

[8] Machida T., Machida N. (2020). Invasion of Pheochromocytoma from the Caudal Vena Cava to the Right Ventricular Cavity in a Dog. Case Rep. Vet. Med..

[9] Takeuchi R., Ishigaki K., Yoshida O., Sakurai N., Terai K., Heishima T., Asano K. (2023). Preemptively planned en bloc resection of an extensive right adrenal pheochromocytoma involving the right hepatic division, caval thrombus and segmental caudal vena cava in a dog with Budd-Chiari-like syndrome. Vet. Med. Sci..

[10] Chen X.P., Qiu F.Z., Wu Z.D., Zhang Z.W., Huang Z.Y., Chen Y.F., Zhang B.X., He S.Q., Zhang W.G. (2006). Effects of location and extension of portal vein tumor thrombus on long-term outcomes of surgical treatment for hepatocellular carcinoma. Ann. Surg. Oncol..

[11] Ito D., Frantz A.M., Modiano J.F. (2014). Canine lymphoma as a comparative model for human non-Hodgkin lymphoma: Recent progress and applications. Vet. Immunol. Immunopathol..

[12] Valli V.E., San Myint M., Barthel A., Bienzle D., Caswell J., Colbatzky F., Durham A., Ehrhart E.J., Johnson Y., Jones C. (2011). Classification of canine malignant lymphomas according to the World Health Organization criteria. Vet. Pathol..

[13] Cleveland M.J., Casale S. (2016). Incidence of malignancy and outcomes for dogs undergoing splenectomy for incidentally detected nonruptured splenic nodules or masses: 105 cases (2009–2013). J. Am. Vet. Med. Assoc..

[14] Seelig D.M., Avery A.C., Ehrhart E.J., Linden M.A. (2016). The Comparative Diagnostic Features of Canine and Human Lymphoma. Vet. Sci..

[15] Kristinsson S.Y., Gridley G., Hoover R.N., Check D., Landgren O. (2014). Long-term risks after splenectomy among 8149 cancer-free American veterans: A cohort study with up to 27 years follow-up. Haematologica.

[16] Chalfon C., Sabattini S., Finotello R., Faroni E., Guerra D., Pisoni L., Ciammaichella L., Vasconi M.E., Annoni M., Marconato L. (2022). Lymphadenectomy improves outcome in dogs with resected Kiupel high-grade cutaneous mast cell tumours and overtly metastatic regional lymph nodes. J. Small Anim. Pract..

[17] Diaz-Vico T., Fernandez-Hevia M., Suarez-Sanchez A., Garcia-Gutierrez C., Mihic-Gongora L., Fernandez-Martinez D., Alvarez-Perez J.A., Otero-Diez J.L., Granero-Trancon J.E., Garcia-Florez L.J. (2021). Complete Mesocolic Excision and D3 Lymphadenectomy versus Conventional Colectomy for Colon Cancer: A Systematic Review and Meta-Analysis. Ann. Surg. Oncol..

[18] Gupta A., Garabetian C., Cologne K., Duldulao M.P. (2024). Complete mesocolic excision and extended lymphadenectomy: Where should we stand?. J. Surg. Oncol..

[19] Ponce F., Marchal T., Magnol J.P., Turinelli V., Ledieu D., Bonnefont C., Pastor M., Delignette M.L., Fournel-Fleury C. (2010). A Morphological Study of 608 Cases of Canine Malignant Lymphoma in France With a Focus on Comparative Similarities Between Canine and Human Lymphoma Morphology. Vet. Pathol..

[20] Kyles A.E., Feldman E.C., De Cock H.E.V., Kass P.H., Mathews K.G., Hardie E.M., Nelson R.W., Ilkiw J.E., Gregory C.R. (2003). Surgical management of adrenal gland tumors with and without associated tumor thrombi in dogs: 40 cases (1994–2001). J. Am. Vet. Med. Assoc..

[21] Lang J.M., Schertel E., Kennedy S., Wilson D., Barnhart M., Danielson B. (2011). Elective and emergency surgical management of adrenal gland tumors: 60 cases (1999–2006). J. Am. Anim. Hosp. Assoc..

[22] Massari F., Nicoli S., Romanelli G., Buracco P., Zini E. (2011). Adrenalectomy in dogs with adrenal gland tumors: 52 cases (2002–2008). Javma-J. Am. Vet. Med. A.

[23] Collivignarelli F., Bianchi A., Paolini A., Vignoli M., Crisi P.E., Falerno I., Bonis A., Rosto M., Tamburro R. (2022). Two-Port Laparoscopic Adrenalectomy in Dogs. Animals.

[24] Farrell M., Singh A., Mayhew P.D., Lillo-Araya F., Massari F., Richardson D., Collier A.J. (2023). Bilateral, single-session, laparoscopic adrenalectomy was associated with favorable outcomes in a cohort of dogs. Javma-J. Am. Vet. Med. A.

[25] Carling T., LaRue M. (2025). Improved and individualized approach to adrenal surgery. Endocr. Relat. Cancer.

[26] van Bokhorst K.L., Galac S., Kooistra H.S., de Grauw J.C., Teske E., Grinwis G.C.M., van Nimwegen S.A. (2023). Laparoscopic vs. open adrenalectomy: Perioperative data and survival analysis in 70 dogs with an adrenal tumor. Front. Vet. Sci..

[27] Rosa C., Schoeman J.P., Dvir E. (2012). Budd-Chiari-Like Syndrome Associated with a Pheochromocytoma Invading the Right Atrium in a Dog. Isr. J. Vet. Med..

[28] Pagani E., Tursi M., Lorenzi C., Tarducci A., Bruno B., Mondino E.C.B., Zanatta R. (2016). Ultrasonographic features of adrenal gland lesions in dogs can aid in diagnosis. Bmc Vet. Res..

[29] Zini E., Nolli S., Ferri F., Massari F., Gerardi G., Nicoli S., Romanelli G., Montinaro V., Trez D., Cavicchioli L. (2019). Pheochromocytoma in Dogs Undergoing Adrenalectomy. Vet. Pathol..

[30] Meuten D.J., Moore F.M., Donovan T.A., Bertram C.A., Klopfleisch R., Foster R.A., Smedley R.C., Dark M.J., Milovancev M., Stromberg P. (2021). International Guidelines for Veterinary Tumor Pathology: A Call to Action. Vet. Pathol..

[31] Hertel B., Bortolami E., Furlanello T., Bertolini G., Cinti F. (2025). Successful venotomy for portal tumor thrombus removal due to pancreatic carcinoma in a dog. Vet. Surg..

